# A Critical Look at Colloid Generation, Stability, and Transport in Redox-Dynamic Environments: Challenges and Perspectives

**DOI:** 10.1021/acsearthspacechem.3c00255

**Published:** 2024-03-22

**Authors:** Eleanor Spielman-Sun, Kristin Boye, Dipankar Dwivedi, Maya Engel, Aaron Thompson, Naresh Kumar, Vincent Noël

**Affiliations:** † Environmental Geochemistry Group at SLAC, Stanford Synchrotron Radiation Lightsource (SSRL), SLAC National Accelerator Laboratory, Menlo Park, California 94025, United States; ‡ Earth and Environmental Sciences Area, Lawrence Berkeley National Laboratory, Berkeley, California 94720, United States; § Department of Soil and Water Sciences, Faculty of Agriculture, Food, and Environment, The Hebrew University of Jerusalem, Rehovot 7610001, Israel; ∥ Department of Crop and Soil Sciences, University of Georgia, Athens, Georgia 30602, United States; ⊥ Soil Chemistry, Wageningen University and Research, Wageningen 6708 PB, The Netherlands

**Keywords:** redox, colloids, colloidal stability, experiment vs model, nutrient and contaminant mobility, colloid transport modeling

## Abstract

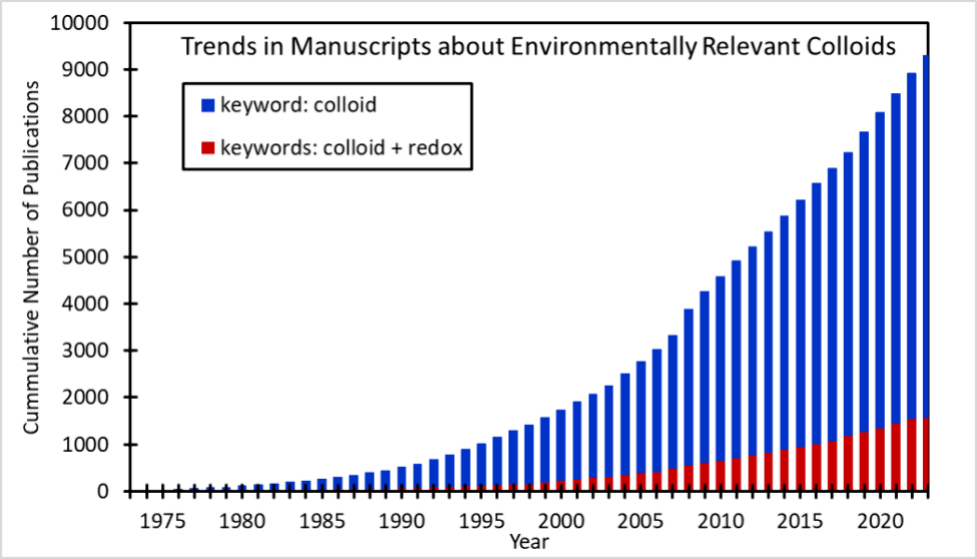

Colloid generation, stability, and transport are important processes that can significantly influence the fate and transport of nutrients and contaminants in environmental systems. Here, we critically review the existing literature on colloids in redox-dynamic environments and summarize the current state of knowledge regarding the mechanisms of colloid generation and the chemical controls over colloidal behavior in such environments. We also identify critical gaps, such as the lack of universally accepted cross-discipline definition and modeling infrastructure that hamper an in-depth understanding of colloid generation, behavior, and transport potential. We propose to go beyond a size-based operational definition of colloids and consider the functional differences between colloids and dissolved species. We argue that to predict colloidal transport in redox-dynamic environments, more empirical data are needed to parametrize and validate models. We propose that colloids are critical components of element budgets in redox-dynamic systems and must urgently be considered in field as well as lab experiments and reactive transport models. We intend to bring further clarity and openness in reporting colloidal measurements and fate to improve consistency. Additionally, we suggest a methodological toolbox for examining impacts of redox dynamics on colloids in field and lab experiments.

## 1 Introduction

The existence of colloids and their role in nutrient and contaminant transport is well recognized in both oxic and anoxic environments. Despite the ubiquity of colloids in environmental systems, defining a “colloid” still remains a challenge. Colloids are broadly defined as particles that are mobile in water but not fully dissolved as solutes. Commonly reported colloids in the environment include clay minerals, metal (oxyhydr)oxides, bacteria, viruses, and organic macromolecules.^1−3^ Colloids exhibit unique properties and behaviors due to their immense intra- and interparticle chemical heterogeneity, making differentiating “colloids” from the conventional “dissolved” vs “solid” phases quite difficult.

Owing to their high specific surface area (SSA) and reactive surface functional group density, colloids are important carriers of critical substances, including organic compounds, macro- and micronutrients, heavy metals, and organic pollutants, and therefore, have influence over ground and surface water quality.^4−10^ The efficiency of colloidal transport relies on the stability of the suspension, with prolonged stability directly facilitating transport.^5^ Colloid-facilitated transport of nutrients and contaminants can sustain aqueous-phase concentrations beyond thermodynamic solubility^11^ and thus, in certain conditions, can enhance the transport of sparingly soluble contaminants.^10,12−16^ Despite these observations, the exact mechanisms of colloid generation, their biogeochemical behavior, and transport, particularly in redox-dynamic environments, remain elusive.

Recurring seasonal wetting and drying of soils and sediments drives redox processes at solid–water interfaces.^17−22^ Wetting (e.g., through rain/snowmelt events) can promote colloidal release and downward transport in both saturated and unsaturated soils,^13,23−26^ as can prolonged inundation.^9^ This may be due to physical dislodging (e.g., shearing, sloughing) by high flow rates and/or desorption induced by changes in water chemistry, which can subsequently lead to changes in colloid composition and rate (e.g., surface charges, ligand concentrations).^9,25,27^ Notably, redox shifts during water table level fluctuations are particularly conducive to the generation/release of colloids.^9,15,28−30^ In anoxic environments, the abundance of colloids may also increase by processes such as reductive dissolution of redox-sensitive (oxyhydr)oxides, leading to the release of other minerals and/or organics as colloids.^9,15,31^ This may occur either directly by dissolution/transformation of the redox-sensitive mineral host phases or indirectly by changing the pH or ionic strength, which triggers dissolution/disassembly and stimulates particle dispersion.^32,33^ Secondary processes may also contribute to colloid formation or release; for example, reductively dissolved Fe(II) can react with sulfide to form FeS colloids,^34−36^ or organics released through reductive mineral dissolution can form chelating complexes with solid-phase, surface-associated elements/minerals to generate suspended organo-mineral colloids.^37−39^ Conversely, the addition of organic matter to the aqueous phase in anoxic environments has also been shown to result in a gradual decrease in colloid abundance by inducing flocculation (colloid aggregation and settling out of suspension).^40^

When conditions switch to oxidizing, dissolved Fe(II), among other redox-sensitive metal(loid)s can oxidize quickly and form colloidal Fe(III) phases that may remain suspended in the aqueous phase because of their small size and/or the presence of organic chelating agents.^40−44^ However, oxidation tends to increase the partitioning of (previously) suspended materials toward the solid-phase and thereby lead to a more or less gradual (i.e., kinetically controlled) decrease in colloid abundance.^45^

Depending on the biogeochemical conditions (e.g., nature of colloids, aqueous-phase composition, and solid-phase properties), colloids have been found to move faster,^46,47^ at the same rate,^26,47^ or slower^10^ than conservative tracers, highlighting the need to develop a better understanding of biogeochemical parameters that influence this transport in porous media. Additionally, the impact of redox conditions on colloid transport will also largely depend on the chemical and physical characteristics of these colloids. With all these dynamic processes, and codependency in colloidal generation and behavior in response to redox-dynamic environmental systems, a useful modeling framework that includes redox-generated colloids and their transport into biogeochemical modeling is critical, but yet to be fully developed. This lack of a modeling tool currently leads to the underestimation of the impact of redox processes on colloid-facilitated transport in the environment, which leads to experimental designs that lack colloidal consideration, feeding back to dissuade researchers from collecting enough empirical data to develop such models in the first place.

Several studies have reviewed the characterization and behavior of colloids in natural systems.^1,3,48−53^ This review specifically focuses on critically evaluating how colloids in natural systems influence and are influenced by redox cycling. With S, Fe, Mn, and organic phases together composing the most ubiquitous redox-sensitive phases in soil, sediment, and water ecosystems, and Mn concentrations being typically lower, the redox transformation of S, Fe, and organic phases and subsequent colloid generation are the most studied colloidal phases in redox-affected ecosystems. As a consequence, several mechanisms underlined in this review that impact the generation, stability, and transport of colloids in redox-affected environments are mainly illustrated using examples focusing on S, Fe, and organic phases. We primarily focus on the definition (Section 2), the mechanisms of colloid generation in redox-dynamic environments (Section 3), the chemical controls over colloidal behavior in redox-dynamic environments (Section 4), the challenges of sampling in redox-dynamic field sites and associated experimental needs in predicting colloidal transport in redox-dynamic environments (Section 5), and the state of colloid transport modeling and associated challenges in incorporating redox chemistry (Section 6). Finally, we highlight the need to consider colloids in regular experimental and measurement plans and in biogeochemical modeling for a better understanding of the role and extent colloids have in elemental cycling and contaminant/nutrient transport. We also make recommendations and suggestions for promising approaches in future research of colloid biogeochemistry.

## 2 What Is a Colloid? The Definition Conundrum

Much debate surrounds the definition of a colloid in environmental systems. Historically, particles have been termed “colloids” based on three general metrics: (i) they are within a certain particle size range, (ii) they remain stable in the aqueous phase for extended periods, and (iii) therefore, they exhibit some potential for mobility/transport. Here, we summarize the existing literature to provide background on the concept of colloids and provide criteria for when and how to use the term “colloid”. For this manuscript, we use the term “aqueous phase” to define a solution that contains dissolved species and suspended particles.

### 2.1 Size Cutoff and Beyond

**Figure 1:**
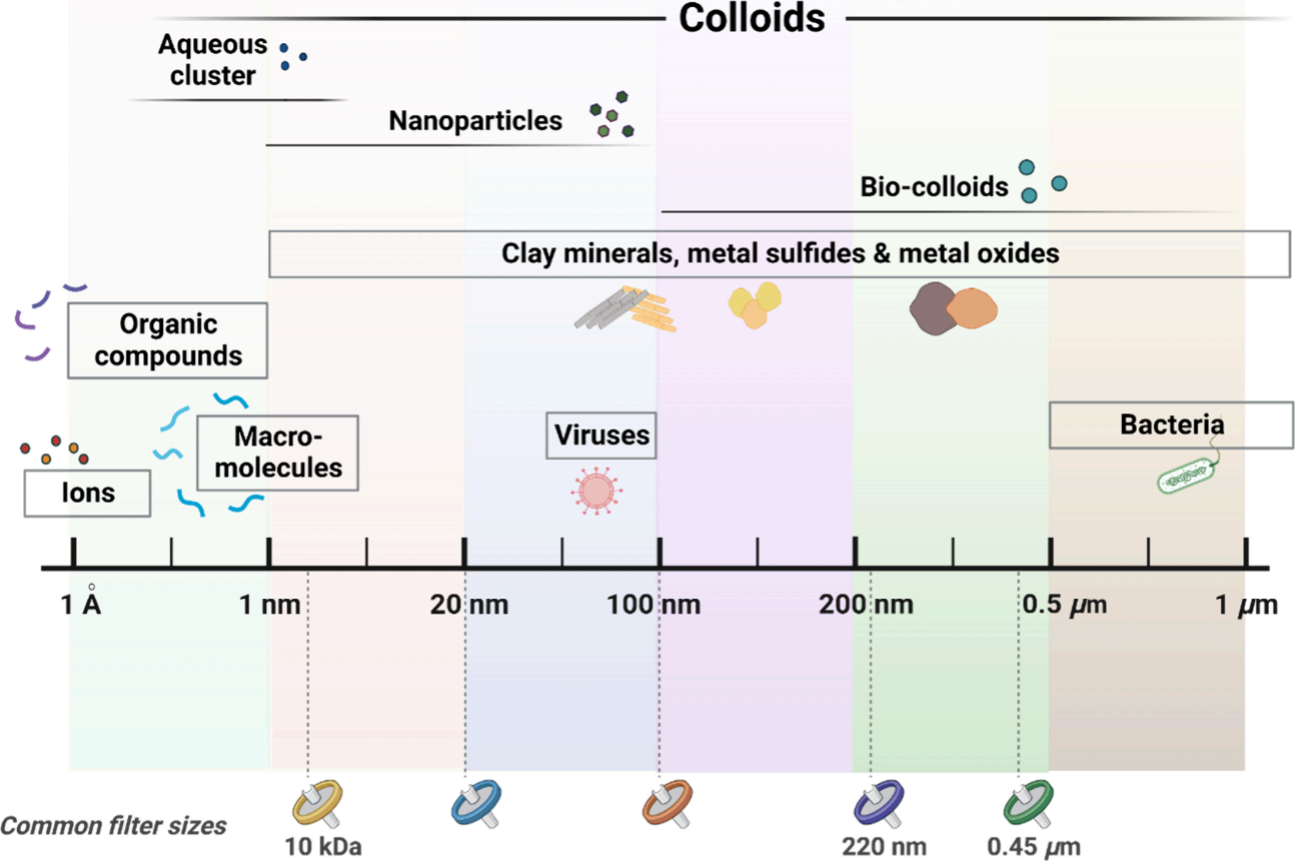
Size range of solutes and environmental particles often considered colloids when between 0.5 nm and 1 μm. Commonly used aqueous sample filter sizes are shown for comparison.

The most common characteristic used to distinguish a colloid from dissolved or suspended particulate materials is its size—a solid particle with at least one dimension between 1 nm and 1 μm is often considered a colloid (Figure 1).^1,30,54^ Although convenient for methodological standardization purposes,^55^ this operational definition is problematic from a biogeochemical point of view, as the functional differences between colloids and dissolved species are not considered. Furthermore, biases may arise due to the use of a specific filter pore size or material,^56^ which can introduce filtration artifacts due to the inclusion/exclusion of specific colloidal particles (as discussed in later in Section 6.1).^57,58^ Within different research disciplines, additional terminology has emerged for colloid subclasses based on functional behavior, but still defined by size, for example, aqueous clusters (0.5–2 nm),^31,59,60^ nanoparticles (1–100 nm),^61−63^ and microbes/biocolloids (100 nm to 1 μm).^64,65^

Aqueous clusters are polynuclear complexes in the aqueous phase that precede the formation of solid nanoparticles.^66^ During particle precipitation at the nucleation stage, an aqueous cluster grows in size, accumulating additional molecular groups in a specific structure. Once the aqueous cluster reaches a critical radius and mass, the structure condensates to form a solid nanoparticle.^66^ Thus, the structure of the aqueous cluster serves as the basis of the structure of the nanoparticle.^67^ For example, FeS aqueous cluster stoichiometry ranges from Fe_2_S_2_ to Fe_150_S_150_, before the first condensed nanoparticle appears.^67^ While aqueous clusters and nanoparticles can overlap in dimensions, particularly during the initial condensation phase, they have distinct physicochemical properties due to their different surface area to volume ratio. Importantly, despite their obvious metastable nature, some aqueous clusters have high stability constants^68,69^ and are resistant to redox transformation and dissociation,^69,70^ which explains their persistence and transport in surface waters.^71^ Due to the ability for these clusters to stay suspended in the aqueous phase for extended periods of time (>30 days; as discussed in Section 4.3.2) and similarity in size, chemical composition, and structure between aqueous cluster and nanoparticles, authors often simply refer to these aqueous clusters as colloids.^72,73^ However, because clusters with minimal stoichiometry can be smaller than 1 nm (up to 0.5 nm),^67^ using a strict size definition for colloids is difficult. For the purposes of this manuscript, we consider “aqueous clusters” to be distinct from “nanoparticles”, but both can still be subcategorized within the broader “colloids” term.

Within the colloidal subclass of inorganic nanoparticles, studies have shown that the particle size has a significant impact on their chemical properties and structure, such as the total free surface energy^74^ and structural strain.^75−78^ The percentage of atoms localized at the surface increases compared to the bulk as the particle diameter decreases, leading to excess surface energy.^79^ Strain-induced lattice contraction is caused by structural stress (the stress applied at the surface of the particle as a result of size reduction) and can significantly affect the particle reactivity.^80−82^ On the other hand, many organic compounds are poorly soluble or even insoluble in water, leading to the formation of organic nanoparticles^83−85^ such as (bio)polymers like lipoproteins, polysaccharides, and polyuronides.^3,86,87^ Overall, size and surface properties (including surface functional groups) of organic colloids drive their unique reactivity and suspension stability in the aqueous phase. Organic nanoparticles are negatively charged colloids, mainly due to the dissociation of hydroxyl and phenolic (∼−OH) groups, as well as carboxylic (−COOH) groups. These colloids are mostly pH-dependent because the Cation Exchange Capacity (CEC) process depends on the replacement of hydrogen; thus, CEC normally increases at higher pH values, becoming highly negative in neutral to alkaline soils.^88^ Because of highly negative charges available on the surface, the organic nanoparticles sorb/complex different positively charged organic and inorganic constituents, thus being able to store essential nutrients and a high rate of contaminants.^3,87^ Additionally, organic nanoparticle colloids can associate with positively charged inorganic nanoparticles, promoting formation of colloids, thus combining the characteristics of organic and inorganic constituents at the nanoscale.^89^

Finally, biocolloids are microbial cells either associated with colloidal assemblages^90^ or present as individual entities, i.e., live organisms (bacteria, archaea, viruses, protozoa), that exhibit transportability in the aqueous phase.^53^ Biocolloids may form by sloughing or detachment of organisms from biofilms or consist of planktonic organisms that exhibit a “nomadic lifestyle”.^24,91^ Notably, as they are living cells, biocolloids do not dissolve/reform (even if they can partially transform (e.g., rupture of cell wall, osmosis) in response to aqueous-phase chemistry such as pH and ionic strength) or even move like abiotic colloids in response to changing conditions. Although biocolloids typically respond to the same parameters as abiotic colloids (e.g., flow rate, ionic strength, pH), they also exhibit specific “biological strategies”, including sporulation (in suboptimal environmental conditions), multiplication (in beneficial environmental conditions), and/or have extracellular structures that allow them to actively attach to specific colloidal/solid-phase matrix surfaces (e.g., by lipopolysaccharides, curli, pili) or move to/from beneficial/detrimental conditions (e.g., by flagella, cilia).^53,65,91^

### 2.2 Colloidal Stability in the Aqueous Phase

Colloids are thermodynamically metastable and, with time, transform to more stable phases. Thus, clusters will have a tendency to condense into solid nanoparticles, and nanoparticles to aggregates. The ability of a colloid to stay suspended in the aqueous phase, termed colloid stability, is thus related to resistance against condensation, aggregation, and chemical reactivity. Atomic and electronic structures at surface and near-surface of colloids vary with their intrinsic physicochemical properties (e.g., size, shape, morphology, surface properties), influencing resistance against dissolution, chemical transformation, and electrostatic and electrosteric repulsive forces that alter aggregation kinetics.^92^ As an example, a modified version of the Kelvin equation predicts solubility dependence on size, stating that as particles get smaller through the nano range of sizes, their solubilities increase exponentially.^93^ However, certain minerals are known to become less soluble as they get smaller in size.^94^ Thus, intrinsic physicochemical properties, such as the size, directly influence the colloidal stability of nanoparticles. Among inorganic nanoparticles, some minerals only exist in the nano size range, such as iron (e.g., ferrihydrite) and manganese (oxyhydr)oxides, that have been defined as nanominerals.^92^ Unlike nanominerals, mineral nanoparticles are minerals that can also exist in larger sizes.^92^ With the mineral nature driving the nanoscale size range of a nanoparticle, one can anticipate a change in the colloidal stability depending on the mineral nature of the nanoparticle.^92^ Finally, coprecipitation of a cluster and nanoparticle, composing colloids, in the presence of trace metal, organic compounds, and nutrients, can influence the intrinsic physicochemical properties of colloids, such as solubility product (as discussed in later in Sections 4.2.1 and 4.3.1).

**Figure 2:**
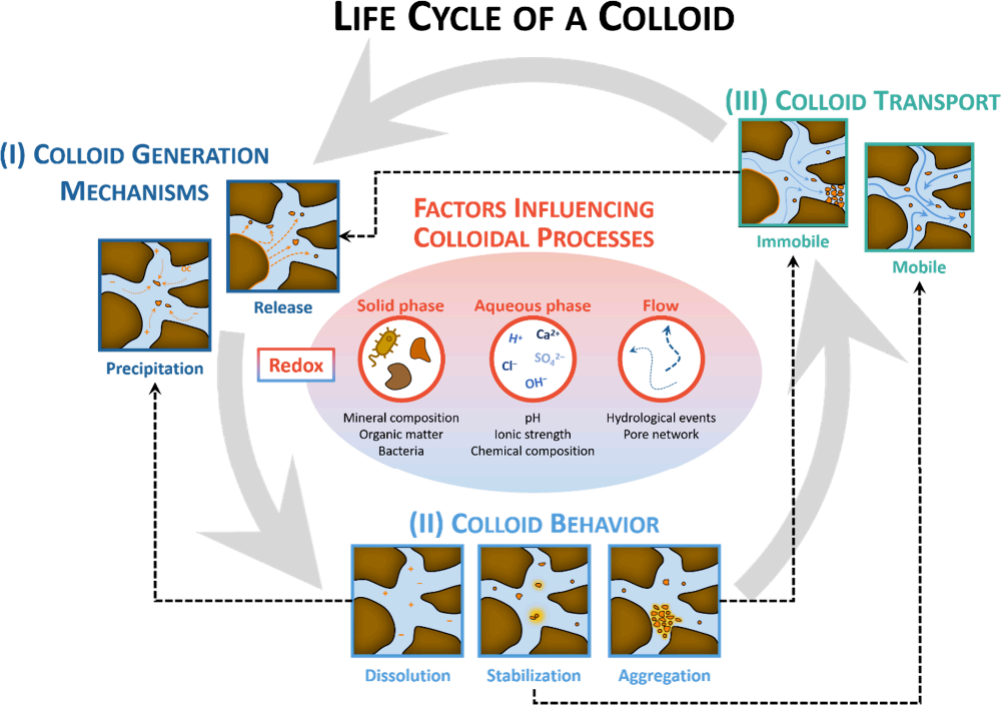
Life cycle of natural colloids in the environment. (I) Colloids can be generated either by release from the solid-phase matrix or by *de novo* precipitation in aqueous phase. (II) Once formed, colloids can undergo dissolution, aggregation, or further stabilization. (III) Colloids, depending on their physicochemical properties, can either remain mobile in aqueous phase or become immobile and attached to or part of the solid- phase matrix.

In addition to intrinsic physicochemical properties, the ability of a particle to stay suspended in the aqueous phase (i.e., colloid stability) is strongly dependent on aqueous-phase chemistry, including pH, ionic strength, ionic composition, and solid/solution ratio (Figure 2). These aqueous-phase compositions affect the magnitude and nature of the colloid’s effective surface charges and thus the interparticle electrostatic repulsion/attraction between suspended colloids/particles, solutes, and the nondispersible solid-phase matrix.^3,27,32,95−97^ Electrostatic and/or steric forces at the colloid surface (and/or the solid-phase matrix) can repel particles from one another and keep colloids in suspension.^3^ In contrast, van der Waals and magnetic dipolar interactions can enhance interparticle attraction (or to the solid-phase matrix),^3^ promoting particle aggregation, flocculation, sorption, and/or physical filtration that can remove colloids from suspension.

Consequently, both the physical and chemical characteristics of colloids are highly sensitive to the conditions of the surrounding aqueous-phase and bulk solid- phase matrix (Figure 2). For example, mineral colloidal suspensions are generally favored at pH values that are far away from the point of zero charge (PZC) of the particles,^98−100^ whereas rapid coagulation is promoted in the vicinity of the PZC.^3,101^ Additionally, aggregation becomes progressively less reversible with time spent at a aqueous-phase pH somewhat near the colloid’s pH_PZC_.^102^ Colloid dispersion is also favored at low ionic strength because increasing ionic strength can cause colloids to reach the minimum concentration of counterions to induce coagulation of colloids, referred to as the critical coagulation concentration (CCC).^103,104^ This process also applies to biocolloids, as observed by Jewett et al.,^105^ who found that decreasing the ionic strength caused the bacterial attachment to decrease in column experiments. Changes in ionic strength, pH, and organic matter concentration/composition can trigger colloid aggregation or dissolution, chemical adsorption or desorption, physical attachment (filtering) or detachment (sloughing), and/or chemical (including redox) transformations.^25,40,97,98,106−109^ These geochemical factors that influence colloidal stability during redox cycling are further discussed in Section 4**.**

### 2.3 Colloid Mobility and Transport

Transport of colloids in a specific soil/sediment/aquatic environment at a given time point is dependent on a complex combination of factors, including the colloid chemical composition,^3,110,111^ the solid-phase matrix composition (e.g., texture, mineralogy),^9,25,35,96,108,109,112^ the aqueous-phase chemistry,^10,27,104,113^ the hydrological flow regime,^25,108,112^ the organic matter concentration and composition,^40,96,114−119^ and the macro-/microecological communities.^110^ Depending on the aforementioned factors, the time for which particles stay suspended in the aqueous phase as colloids can vary from seconds or hours to months or years.^31,99^ Thus, colloidal abundance is properly conceptualized as dynamic over both space and time—especially in redox-dynamic environments. These are further discussed in Section 4**.**

### 2.4 Guidelines for Using the Term “Colloids”

We propose the following guidelines that will facilitate future comparisons between colloid-related studies, while recognizing the methodological constraints involved. (1) The size range (or other criteria) used to define a colloid should be flexible, but clearly stated; a wider size range (0.5 nm to 1000 nm) is preferable, or one that is tailored to the substance(s)/element(s) of interest, i.e., the “function” should take precedence over operational partitioning to the extent possible. (2) The time frame at which colloid stability/mobility is monitored/considered should be duly noted, in order to assess the environmental persistence and potential influence of the colloids.

## 3 Mechanisms of Colloid Generation in Redox-Dynamic Environments

Redox processes, such as oxidative dissolution of sulfide minerals and Fe redox transformation,^28,101,120−123^ induce changes in aqueous-phase parameters (e.g., pH, ionic strength, and ionic composition) and chemical, organic, and mineral transformation of the solid-phase matrix that can generate (in)organic colloids, mobilizing nutrients and contaminants. NOM complexation/coating can also play a vital role in the liberation of metal(loid)s from the solid-phase matrix and their stabilization as colloids in suspension.^3,96,97,124^ While the generation of Fe-(oxyhydr)oxide colloids under oxic conditions has been extensively studied,^(e.g.,^^37,41,45,125^) significantly less focus has been placed on colloid generation in redox dynamic environments. Additionally, though recent studies have reported colloid formation in anoxic environments,^31,63^ including soil and sediments, the exact mechanism, kinetics, and fate are still elusive.

### 3.1 Formation of Colloids in Anoxic Environments

Under anoxic conditions, mineral precipitation, change in chemical speciation, and organic complexation are widely understood to transform and remove nutrients (e.g., N, S, and P)^15,126,127^ and/or contaminants (e.g., divalent heavy metals,^128−130^ Cr,^131^ As,^132−134^ Sb,^135,136^ Se,^137^ U,^138,139^ V, Tc,^140^ and halogenated organic compounds^141,142^) from pore- and groundwater. Paradoxically, colloids may readily transport nutrients and contaminants traditionally thought to be immobilized under anoxic conditions. In this section, we examine the mechanisms behind this seemingly paradoxical behavior and highlight its importance in redox-dynamic environments.

#### 3.1.1 Colloid *In Situ* Release Due to Reductive Dissolution of the Solid-Phase Matrix

In general, reductive dissolution can promote colloid dispersion by dissolving the solid-phase matrix that holds the aggregated particles together.^143^ Fe minerals, especially nanoparticulate and short-range-ordered (SRO) Fe phases,^144^ are commonly cited as important binding agents for larger soil aggregates.^145^ In natural systems, (bio)reduction of these SRO Fe phases may lead to *in situ* release of colloids.^146^ Henderson et al.^15^ and Buettner et al.^39^ showed that reductive dissolution of Fe(III)-cements could release P-bearing and C-bearing colloids, and Tadanier et al.^143^ demonstrated that bioreduction of Fe-(oxyhydr)oxide aggregates can trigger the formation of nanometer-sized As-bearing Fe(III)-colloids. In all of these cases, the transportability of P, NOM, and As was increased as a result of colloidal *in situ* release generated from reductive dissolution.

Reductive dissolution of Fe(III)-(oxyhydr)oxides often triggers an increase in pH (via OH^–^ release) that can independently drive colloid *in situ* release due to (i) the solid-phase matrix dissolution and (ii) electrostatic repulsion between like-charged particles due to change in surface charge intensity by virtue of changes in pH.^28,147^ Several studies have attempted to untangle the effect of reductive dissolution of Fe(III)-(oxyhydr)oxides from any associated pH increase, but the literature shows contradictory conclusions. For example, De-Campos et al.^148^ showed that Fe reduction decreased soil aggregate stability to a greater extent than changes in aqueous-phase pH alone, while Grybos et al.^147,149^ in a series of studies observed a greater *in situ* release of NOM and metal(loid)s from clay minerals due to aqueous-phase pH change than from the reduction of Mn and Fe-(oxyhydr)oxides alone in wetland soil.

#### 3.1.2 Generation of Sulfurized Metal(loid) Colloids

Under sulfate-reducing conditions, a metal–aqueous sulfide interaction first generates small aqueous complexes, such as aqueous clusters,^66^ preceding the formation of nanoparticles^150^ (see Section 2.1). The thermodynamic and kinetic stability of these aqueous clusters against condensation into nanoparticles suggests their colloidal transport can remain relevant in sulfidic environments for extended periods.^66^ Recently, Noël et al.^31^ showed reductive dissolution of ferrihydrite (poorly crystallized Fe(III)-(oxyhydr)oxides) by aqueous sulfide generates FeS colloidal aqueous clusters. These colloidal clusters, defined operationally in terms of their voltammetric characteristics, have been previously detected in many natural anoxic aqueous and sedimentary environments, including anoxic lake waters and porewaters^151,152^ and sulfidic waters of bays, marshes, hydrothermal vents, and sedimentary porewaters.^153−155^ Rickard and Morse^150^ suggested that rapidly forming metal sulfide colloidal aqueous clusters also exist for Cu, Zn, Ag, and Pb and can make up a substantial fraction of the sulfide and metal budget in anoxic environments. Additionally, the authors claim that colloidal aqueous clusters of FeS, ZnS, and CuS could constitute a major fraction of the dissolved metal load in anoxic oceanic, sedimentary, freshwater, and deep ocean vent environments, supporting the idea that divalent metals do not necessarily always settle under sulfidic conditions but rather can be significantly transported in anoxic environmental systems.

Condensation of these metal sulfide clusters can also produce nanoparticulate colloids by homogeneous precipitation^156^ or by being templated on bacterial membranes.^157^ For example, Kumar et al.^158^ reported that As was transported under sulfate reducing conditions by FeS colloidal nanoparticles. Additionally, Weber et al.^63^ demonstrated that sulfate reduction resulted in the release of Cd and Pb from a contaminated soil as either <50 nm Cu-rich sulfide nanoparticulate colloids or as associated with bacterial membrane biocolloids, which can be transported with advective flow.^159^ Further, finely dispersed sulfide colloids, generally negatively charged at pH > 4,^160^ may resist aggregation and deposition,^161^ as observed for dispersed Cu_*x*_S and CdS colloids in flooded agricultural soils.^162^ Such stable metal sulfide colloids may serve as a mobile carrier phase enhancing the transport of chalcophile nutrients and contaminants by colloid-facilitated transport.

The extent to which the metal-sulfide colloids are formed is controlled by the solubility of the corresponding metal sulfides and limited by the availability of dissolved sulfide.^17,66^ The rates of microbial sulfate reduction and sulfate concentration are thus key parameters controlling the generation rate and the composition of metal-sulfide colloids. For instance, Xu et al.^163^ observed that flooded soil samples with higher sulfate concentrations had greater and more rapid generation of metal colloids with the fraction of colloidal Cu_*x*_S vs Cu(0) increasing with higher sulfate content. Similarly, Noël et al.^31^ showed that the rate of sulfidation (directly driven by the S(−II)/Fe(III) ratio) of ferrihydrite aggregates led to a mixture of ferrihydrite/FeS colloids at a very low sulfidation (S(−II)/Fe(III) ratio of 0.1), while complete conversion to FeS colloids was observed at a higher sulfidation (S(−II)/Fe(III) ratio of 0.5).

#### 3.1.3 Anoxia-Induced *In Situ* Release of Colloidal Natural Organic Matter

Specifically, under anoxic conditions, there are three main proposed mechanisms for the *in situ* release of colloidal NOM (Figure 2). First, minerals undergo reductive dissolution, which subsequently releases any surface-bound NOM.^40^ More specifically, as soils become anoxic, Mn- or Fe-(oxyhydr)oxide minerals, which often contain adsorbed or coprecipitated NOM^164,165^ or which are present as binding agents or cements, undergo reductive dissolution and release NOM,^29,39,166,167^ often still in colloidal co-association with relatively more reduction-resistant minerals, such as clay minerals^168−170^ and silicates.^29^ Second, anoxia-induced changes in aqueous-phase pH and ionic strength can alter mineral surface charge and ionic composition such that NOM-associated colloids are desorbed or dispersed.^32,39,147^ Third, microbial respiration products have also been shown to produce colloidal NOM under anoxic conditions.^171,172^ Once released, this reduced colloidal NOM can reduce redox-active metal(loids)^38^ and/or form stable metal(loid)-NOM complexes,^173,174^ thus potentially enhancing the mobility of these contaminants in subsurface environments.

### 3.2 Formation of Colloids in Oxidizing Environments

When conditions change back from anoxic to oxic, reduced forms can oxidize, releasing metal(loid)s, organics, and nutrients to pore, ground, and surface waters which may also (co)precipitate as/with (oxyhydr)oxide colloids.^41^

#### 3.2.1 Generation of Colloids via Iron Oxidation

In pore, ground, and surface water, both the transport of dissolved Fe(II)- and Fe(II)-colloids into more oxic environments and the introduction of O_2_ into anoxic zones can trigger the oxidation of Fe(II) to Fe(III), resulting in the formation of Fe(III)-rich colloids.^37,125^ From dissolved Fe(II), these Fe(III) phases begin as small aqueous clusters of octahedral Fe(O, OH, OH_2_)_6_ units and grow into larger clusters over time, eventually reaching the size of nanoparticles (i.e., >1 nm). The driving force for crystal growth and/or aggregation of these nanoparticles is the decrease in surface energy.^175^ However, for ferrihydrite, and possibly other Fe phases, the surface energy appears to be low enough that these nanoparticles are more stable in the nano size range and do not grow further^176,177^ and are defined as nanominerals.^92^ Because ferrihydrite nanominerals consistently remain in the nano size range and thus in suspension,^178^ they are among the most ubiquitous natural inorganic colloids.^179^ Further discussion on the conversion of metastable ferrihydrite colloids into more stable Fe(III)-(hydroxy)oxide colloids can be found later in Section 4.3.1). During the oxidation processes, nutrients and contaminant metal(loid)s may associate with the Fe(III)-(oxyhydr)oxide colloids via (re)/coprecipitation or surface complexation.^180−183^ In particular, mobile ferrihydrite colloids commonly serve as geochemical vectors, facilitating the transport of a range of contaminants, such as As,^184,185^ Pb,^183^ and nutrients such as phosphate^186−189^ within aqueous environments at anoxic–oxic interfaces.

Overall, oxidation increases the nucleation of (oxyhydr)oxide colloids, and even elements inert to redox changes, such as Al, can associate with (oxyhydr)oxide nanosized colloids to form larger colloids.^169^ The extent to which the metal-(oxyhydr)oxide colloids are formed is controlled by the solubility of the corresponding metal-(oxyhydr)oxides and limited by the availability of the metal cation. As an example, Liang et al.^41^ studied the dynamics of dissolved, colloidal, and deposited Fe phases using a forced-gradient field experiment with the injection of oxygenated water with a high NOM concentration into a sandy aquifer. Under the increased dissolved oxygen (DO; 2 mg L^–1^) conditions, Fe(II) oxygenation was rapid, resulting in the formation of Fe(III)-(oxyhydr)oxide colloids. However, when DO was low (0.2 mg L^–1^), oxidation was much slower, promoting the formation of Fe-organic dissolved complexes (up to 80% of Fe(III) was in the dissolved phase) and subsequently limiting the Fe(III)-(oxyhydr)oxide colloid formation. These dissolved complexes could significantly limit the availability of metals to form metal-(oxyhydr)oxide colloids. However, in natural conditions, Fe(III)-organic dissolved complexes can sorb at the surface of (oxyhydr)oxide colloids, forming Fe(III)-organic-(oxyhydr)oxide colloids.

#### 3.2.2 Generation of Colloidal Natural Organic Matter in Oxic Systems

In pore, ground, and surface waters, colloids provide large surface areas to which NOM can sorb. Overall, because NOM has a strong affinity for freshly precipitating Fe(III) relative to Fe(II),^190^ associations of Fe(III)-colloids with NOM are abundant.^169,191,192^ Indeed, at typical natural pH values of 4–7, a fraction of NOM is negatively charged, while oxidized Fe(III)-(oxyhydr)oxide colloid surfaces, such as ferrihydrite, carry a net positive charge, creating favorable conditions for their electrostatic interaction.^193−195^ As an example, Riedel et al.^164^ observed the precipitation of Fe-(oxyhydr)oxides in peatland samples at the anoxic/oxic interface, which subsequently coagulated with plant-derived and pyrogenic NOM. By extension, Lyvén et al.^181^ found that Fe(III)-(oxyhydr)oxide colloids in river waters are present as small nanosized colloids closely associated with humic-type NOM. The formation of these Fe(III)-(oxyhydr)oxide-NOM colloids enhances available surface area and, hence, available sorption capacity, potentially promoting the adsorption of nutrients and contaminants.^196^ NOM interactions may also promote the dissolution of the Fe(III) colloids into Fe(III)-NOM dissolved complexes (usually defined in the literature to be <1 nm).^197,198^ Ultimately, there is a fine line between dissolved Fe-NOM and colloidal Fe-NOM, further emphasizing the challenges in identifying a colloid’s potential to react and transport. Because Fe-NOM interactions are common in aqueous systems,^183^ they are an important potential transport vector for nutrients and contaminants such as heavy metals.

### 3.3 *In Situ* Release of Biocolloids

Within redox transition zones of subsurface environments, redox-sensitive elements mediate electron-transfer reactions within living cells and subsequently undergo redox transformations linked to microbial energy generation through its utilization as a source of chemical energy or as an electron acceptor for anaerobic respiration.^199^ Thus, redox-sensitive nutrients and contaminants are intimately linked to biocolloids (i.e., viruses, bacteria, spores, etc.), promoting their transports. For example, bacteria dispersed in the anoxic porewater can transport metal(loid)s and induce biomineralization. Xia et al.^200^ observed that the dynamics of Cu in flooded agricultural soils were dominated by microbe-associated colloids and consisted mostly of Cu(0) biomineralization and subsequent sulfidation. This recent study corroborates observations from Weber et al.,^17^ which suggest Cu(0)-bacteria colloids forming by disproportionation of Cu(I) are released by Cu-stressed bacteria to maintain Cu homeostasis.^201,202^ Under oxic conditions, more favorable for microbial growth, biocolloids can release organic carbon that can alter mineral surface properties and colloid aggregation.^203^

Notably, most of the literature regarding biocolloids in the terrestrial subsurface is focused on pathogens that enter the environment from sewage, landfills, and other wastewater/fecal contamination pathways,^53,65^ or organisms that are intentionally introduced for bioremediation purposes.^26,53^ Thus, there is limited information available on natural biocolloids in redox-dynamic environments.^24^

## 4 Geochemical Controls over Colloidal Composition, Stability against Aggregation, and Behavior across Redox-Dynamic Zones

Colloidal suspensions are inherently metastable systems because the stability of colloids is primarily dependent on their ability to resist aggregation and chemical transformation, which are controlled by physical and chemical interactions that readily change.^104,113,114,204−206^ During aggregation, colloids dispersed in the aqueous phase assemble together, leading to settling and decreased transport.^207^

Redox-dynamics and aqueous-phase chemical composition (e.g., ionic strength and composition, pH, presence of NOM or other reactive ions) influence intrinsic physicochemical properties (e.g., size, shape, morphology, strain, aging, surface area, chemistry, superparamagnetism, and surface coatings) of redox-generated colloids, which, retrospectively, affect aggregation.^35,97,209,104,113−118,208^ In addition to the resistance to aggregation, persistence and chemical stabilization of redox-sensitive colloids and sparingly soluble contaminants can occur, even when exposed to variable redox conditions.^66,71,173,192^

### 4.1 Influence of Aqueous Phase Chemistry on Colloidal Stability against Aggregation in Redox-Dynamic Environments

The amount of colloid dispersion is strongly dependent on aqueous-phase pH, ionic strength, and ionic composition because these parameters affect the magnitude and nature of colloid and solid matrix surface charges and, as a result, interparticle attractive and repulsive forces. As discussed in Section 3, the redox processes directly impacting the structure/composition of colloids influence the stability of colloids against aggregation driven by these aqueous-phase chemical parameters. Retrospectively, the aqueous-phase chemical parameters are also affected by redox mechanisms, such as oxidative dissolution of sulfide minerals and Fe mineral redox transformations.^28,120−123^ Thus, redox processes can influence the stability of colloids against aggregation by directly impacting their structure/composition or indirectly by changing the aqueous-phase chemistry (e.g., change in pH and ion composition).^32,95,96^ Additionally, redox-driven biotic or abiotic transfer of electrons may impact surface properties of colloids,^106,210^ likely influencing their surface charges and binding environment. However, as this phenomenon is mainly described for engineered nanoparticles (e.g., Ag(0),^211^ CuO,^212^ zerovalent Fe,^213^ and graphene^214^), the influence of redox processes on the stability of natural colloids is largely overlooked.^106^

#### 4.1.1 Influence of Aqueous Phase pH on Redox-Generated Colloids

As discussed in Section 2.2, mineral colloidal suspensions are favored at pH values far away from their PZC. In redox-dynamic environments, due to protonation–deprotonation reactions, pH can change *in situ* that can influence the colloidal stability.^28^ Under sustained reducing conditions mineralogy can also change, influencing the stability and reactivity of colloids that are generated. For instance, the low pH_PZC_ values of FeS colloids suggests colloid surfaces are negatively charged at pH values typical of naturally reduced zones (pH values of 5–9), resulting in higher interparticle repulsion and, consequently, lower colloid aggregation.^31,215,216^ In contrast, the pH of most natural pore, ground, and surface waters is in the same range as the pH_PZC_ values of ferrihydrite colloids (∼7.5–7.9),^217^ thus resulting in rapid coagulation of ferrihydrite-based colloids in natural systems. Thus, redox dynamics can periodically affect structure/composition of colloids (i.e., intrinsic physicochemical properties) and consequently impact their stability against pH-driven aggregation.

#### 4.1.2 Influence of Ionic Strength and Composition on Redox-Generated Colloids

As discussed in Section 2.2, colloid dispersion is favored at ionic strengths below the colloid’s CCC.^103,104^ Similarly to oxic conditions, in reducing environments, recent studies have shown that higher ionic strength inhibits the generation of FeS colloids during ferrihydrite sulfidation.^31^ Similarly, column studies on the dispersion of Hg sulfide from mine tailings under anoxic conditions imply that sulfide colloids are only mobile at low ionic strengths.^218,219^ Additionally, the biotic or abiotic transfer of electrons driven by redox processes can affect localized ionic strength and pH in the immediate and confined surroundings (not necessarily representative of the bulk) that may lead to colloidal behavior different than that initially expected.

In addition to ionic strength, the ionic composition also impacts colloid stability in suspension.^3,10,196^ The newly formed or released colloids in response to redox fluctuations (see Section 3) are directly influenced by aqueous-phase ionic composition. Thus, nutrient (e.g., NOM, Si, S, P) and/or trace metal(loid) incorporation into the mineral structure or binding to the surface of colloids may change the intrinsic physicochemical properties, stabilizing their suspension and resistance to chemical (e.g., redox) transformations.^220^

#### 4.1.3 Influence of Redox Reactions on Surface Charge

In anoxic conditions, reductive dissolution of ferrihydrite and the ensuing reaction products change surface properties of colloidal ferrihydrite.^28,34^ For example, under sulfate reducing conditions, the partial reductive dissolution of colloidal ferrihydrite promotes the formation and sorption of negatively charged S(0) colloids on the surfaces of the remaining positively charged ferrihydrite, which intensifies the electrostatic attractions between oppositely charged regions on adjacent ferrihydrite surfaces, thus decreasing their CCC, and overall zeta potential of ferrihydrite colloids.^106^ This process accelerates as sulfide concentration increases and further accelerates ferrihydrite aggregation and/or retention in solid matrices. Thus, Liao et al.^34^ concluded that mildly sulfidic anoxic porewater (7.8–46.9 μM sulfide) can significantly decrease the transport capacity of ferrihydrite colloids. However, aggregation of ferrihydrite colloids in mildly sulfidic anoxic porewater is dependent on the physical properties of the ferrihydrite colloids. Indeed, He et al.^106^ showed that aggregation rates could inversely decrease (increasing the colloidal stability) with increasing dissolved sulfide concentration (from 156.3 to 312.5 μM) for the ferrihydrite colloids with higher hydrodynamic diameter. Finally, to date, stability between reduced colloids, such as metal-sulfide colloids, upon mild oxidative diffusion has not yet been thoroughly investigated to our knowledge.^106^

### 4.2 Influence of Natural Organic Matter on Metal(loid) Colloidal Chemical Composition and Stability against Aggregation

NOM coatings on inorganic redox-generated colloids can strongly influence intrinsic physicochemical properties of colloids (e.g., size, shape, morphology, surface properties) and particle surface charge characteristics, inducing electrostatic and electrosteric repulsive forces that alter coagulation kinetics.^4,124,221−228^ Instead of aggregating/settling, redox-generated inorganic colloids can remain mobile due to interactions with NOM. Additionally, redox conditions affect the oxidation states of the redox-active elements, impacting their affinity toward the solid-phase matrix. Complexation with NOM can stabilize metal(loid)s in their less soluble oxidation state as aqueous solutes (usually defined by the community to be <1 nm) or colloids (usually defined by the community to be >1 nm; e.g., Fe(III)-NOM colloids) inhibiting their retention in a solid-phase matrix via adsorption or precipitation.

#### 4.2.1 Impact on Strain and Size of Redox-Generated Colloids

Strain (mineral structure change resulting in response to a stress) has been shown to be affected by surface chemistry and, in particular, by the strength of surface–ligand interactions.^229^ As an example, Le Bars et al.^230^ showed that thiol functional groups can bind strongly to the nano-ZnS surface, reducing the internal strain regardless of particle size. The authors suggested that this strain release could result from a mechanism similar to that reported by Zhang et al.,^231^ that is, a decrease in internal energy and an increase in crystallinity induced by the interaction between water and the ZnS surface. This alteration of strain may explain the transformation of Fe-(oxyhydr)oxides into more^232^ or less crystalline phases^233^ upon exposure to redox fluctuations.

Based on nucleation and growth principles,^234^ organic ligand binding to the surface atoms during colloid formation can inhibit growth by blocking growth sites and, consequently, favor the formation of smaller colloids or aqueous complexes compared to organic-ligand-free systems. Also, if the surface energy decreases as a consequence of organic ligand binding, the system is thermodynamically more favorable for smaller crystal size colloids (reduced critical nucleus size) than an organic-ligand-free system. For example, the nano-ZnS synthesized in the presence of organic molecules containing a thiol group, such as cysteine, exhibits both smaller average crystallite domain sizes and higher strain than the nano-ZnS synthesized under the same conditions in the absence of cysteine.^230^ However, no significant effect on crystallite domain size was observed for laboratory-synthesized nano-ZnS formed in the presence of serine, acetate, and histidine, which highlights that the impact on the colloid crystal growth is dependent on the binding energy of specific organic ligands on the colloid surface.^235^ Thus, the impact of binding organics on redox-generated colloid strain and size is directly dependent on the organic ligand functionality.^230,231,235^

#### 4.2.2 Interplay between NOM and Metal(loid)s in Redox-Dynamic Environments

NOM (including organic ligands) typically has strong affinity for various metal(loid)s and redox-sensitive elements that contributes toward colloidal stability (due to negative surface charge exerted by NOM complexation), for example, of Zn sulfide,^236−240^ Cd sulfide,^241^ Hg sulfide,^242,243^ Cu sulfide,^161^ and Fe sulfide and (oxyhydr)oxide^31,39^ in aquatic environments.

As discussed in Section 3, NOM and Fe are strongly linked. Acidic functional groups (carboxylic, phenolic, carbonyl) on NOM generally favor the formation of surface complexes on the Fe–OH sites of Fe-(oxyhydr)oxides via chemisorption^244^ and promote the stability of Fe(III)-(oxyhydr)oxide colloids.^117,226,245,246^ In particular, Fe-NOM colloids are ubiquitous at anoxic–oxic interfaces and have been extensively studied.^39,41,164,165,247,248^ In this coassociation, the ratio of NOM to Fe can impact the size and stability of these Fe-NOM colloids. Liao et al.^37^ observed that as the C/Fe molar ratio increased, the truly dissolved Fe(II) (as defined by the authors to be <1 nm) decreased 7-fold and the colloidal Fe(II) (1–200 nm) increased accordingly. The C/Fe ratio can also influence colloidal behavior; the authors also observed the aggregation of HA-Fe colloids under both anoxic and oxic conditions decreased with increasing C/Fe molar ratio from 1.6 to 23.3 and the deposition kinetics of Fe(II)-HA colloids under anoxic conditions were slower than those of Fe(III)-HA colloids under oxic conditions.^37^ Liao et al. also observed that the transport of ferrihydrite colloids was substantially lower in the presence of reduced HA than in the presence of oxidized HA, which the authors attributed to enhanced electrostatic and steric stabilization.^249^ Finally, coating of magnetite with HA and phosphatidylcholine was also reported to affect interparticle electrostatic interactions and the colloidal behavior of nanosized magnetite colloids.^250^

The formation of stable colloidal Cr(III)-NOM (10 kDa to 220 nm) upon Cr(VI) reduction by reduced NOM under organic-rich anoxic conditions has been reported.^38^ These colloids are relatively stable in pore, ground, and surface waters of low electrolyte concentrations and are readily transported through porous media.^251^ Colloidal Fe-NOM can also influence Cr reduction. For example, Buerge and Hug^252^ observed the formation of aqueous Cr(III)-NOM-Fe(III) complexes (<450 nm) after Cr(VI) reduction by Fe(II) in organic-rich waters. Pan et al.^253^ observed the formation of stable colloids (1—200 nm) composed of Cr(III), NOM, and Fe(III) during Cr(VI) removal via electrocoagulation at pH 8.0, but below pH 6.0 they aggregated and settled out of the aqueous phase. Upon reaction of Cr(VI) with Fe(II)-HA colloids under anoxic conditions, Cr was completely reduced and formed highly stable Cr(III)-HA-Fe colloids that persisted for at least 20 days, without substantial changes in particle size.^254,255^

Mn is considered to be predominantly present as soluble Mn(II) in anoxic environments and in particulate form (Mn(III/IV)) in oxic environments, but several studies have also observed Mn(III) colloids in anoxic environments.^173,256−258^ Due to the strong affinity of Mn(III) with NOM, up to 90% of the Mn(III) in anoxic systems can be in colloidal form, and this fraction can increase with increasing molar C/Mn ratios.^173^

### 4.3 Persistence, Chemical Preservation, and Transport of Redox-Sensitive Colloids

Pore, ground, and surface water mixing at oxic–anoxic interfaces, or seasonal hydrologic fluctuations, or colloidal transport can expose colloids to redox conditions different from those of their formation. Once exposed to different redox environments, redox-sensitive colloids could play an important role in biogeochemical processes of the surrounding environment. For example, sulfidic colloids exposed to oxidizing environments and oxidized colloids exposed to reducing environments promote electron shuttling that influences geochemical processes and microbial activities.^67,172,259^ Kumar et al.^260^ concluded that organic colloid transport from reducing conditions could influence the spatial extent of biotic and abiotic sulfidic conditions downstream in otherwise oxic aquifers, acting as a vector for biogeochemical reactivity. Persistence and chemical stabilization of redox-sensitive colloids, however, inhibit their redox-driven reactivity with the surrounding environment. Surprisingly, studies have observed the persistence of sulfidic colloids in oxidizing environments^66,261^ and oxidized colloids in reducing environments.^192,262,263^ For these (oxyhydr)oxide and sulfide colloids to exist and transport across oxic and anoxic zones and interfaces, continuous formation and/or dissolution must take place, or they need to be chemically/kinetically stabilized against redox-induced transformation. Thus, redox cycling needs to be considered within the overall theoretical framework for the stability of colloids and their environmental impacts. Some of these factors are discussed below.

#### 4.3.1 Colloid Chemical Stabilization Driven by Mineral Transformations

At the nanoscale, three factors compete to stabilize a given polymorph: enthalpy of polymorphic transition, surface enthalpy, and enthalpy of hydration. In general, the metastable polymorphs of coarse particles have lower surface energies, leading to crossovers in phase stability as the particle size decreases.^92^ This provides a thermodynamic explanation of why nanoparticulate oxides often crystallize as one polymorph, whereas a different polymorph is exhibited in coarser-grained material.^92^ Because a different polymorph exhibits different particle sizes, as well as different crystal structure and specific surface area, conversion from a thermodynamically metastable polymorph colloid to a more stable one can significantly modify colloidal stability.^250,264,265^ For instance, thermodynamically metastable ferrihydrite colloids could transform into crystalline Fe colloids (i.e., goethite, magnetite, and hematite) that are more thermodynamically stable against redox changes. While this transformation is mainly dependent on pH, temperature, Eh, and other dissolved ions, ferrihydrite is highly insoluble at circumneutral pH and the conversion to more thermodynamically stable Fe-(hydroxy)oxide colloids in reducing environments can only proceed in the presence of a catalyst such as Fe(II)_aq_.^266−270^ Because Fe(II)_aq_ is stable and accumulates in high concentration under anoxic nonsulfidic conditions, this transformation pathway can play an important role in the formation of more thermodynamically stable Fe-(hydroxy)oxide colloids at redox transition zones. Thompson et al.^232^ showed that redox oscillations resulted in the transformation of SRO Fe-(oxyhydr)oxides (e.g., ferrihydrite and nanogoethite) into more crystalline Fe-(oxyhydr)oxides (goethite and hematite) in the bulk soil. If this is also true for colloids, the release of SRO Fe colloids and their eventual transformation might result in a different crystal structure and specific surface area of resultant colloids, hence, different behavior and transport potential. Goethite and hematite colloids are expected to resist reductive dissolution longer than ferrihydrite colloids based on reactions of flocculated phases, but this requires further exploration.^271^

In addition to mineral transformation (dissolution/precipitation), clusters and nanoparticles composing newly generated colloids can coprecipitate in the presence of organic, nutrient, and trace metal, impacting the thermodynamic metastability of these colloids. As an example, in the presence of dissolved PO_4_ and As, Fe-colloids coprecipitate as Fe[(OH)_3_,PO_4_]·*n*H_2_O and Fe[(OH)_3_,AsO_4_,PO_4_]·*n*H_2_O, respectively. These nanophases, similar to mixed, metastable nanophases found in certain natural sedimentary environments, do not have bulk counterparts or known solubilities. They are distinct from ferrihydrite or lepidocrocite with sorbed PO_4_ and AsO_4_ and by definition will exhibit different metastability than ferrihydrite or lepidocrocite colloids, which would precipitate in the absence of PO_4_ and As impurities.^272^

#### 4.3.2 Persistence of Sulfidic Colloids in Oxidizing Environments

In sulfidic environments, the generation of sulfide colloids has been proposed to bind and mobilize nutrients and contaminants, particularly chalcophile metals (e.g., Cu, Zn, Cd, Hg, Pb, Sb).^63,72,161,240,273−275^ Thermodynamic and kinetic considerations suggest that metal sulfide colloidal clusters exhibit high stability in oxic aqueous environments,^66^ are remarkably resistant to oxidation, and have been found to contribute to nutrient and contaminant transport in rivers.^71^ Similarly, submicrometer-sized ZnS colloids^261^ have previously been identified in oxic river water. For example, freshly prepared Zn sulfide clusters were found to have a half-life of more than 30 days in the laboratory (Luther et al., Rozan et al., and Vazquez et al.),^69−71^ whereas, in water samples the natural Zn and Cu sulfide clusters (of unknown age) were found to persist after collection for an additional 30 and 42 days, respectively.^276^

In field samples, total metal data obtained from selected acidification or separation experiments,^71,277−279^ pseudovoltammetry measured metal concentrations,^280−282^ and electrochemical or gas chromatographic methods^278,283−285^ suggest that aqueous metal sulfide clusters can complex up to 90–100% of metals in sewage treatment plant waters^71,286^ and oxic waters of rivers, lakes, and the ocean. Consequently, these aqueous clusters can potentially account for a significant fraction of total metal load in oxic pore, ground, and surface waters.^66^

#### 4.3.3 Persistence of Oxidized Colloids in Sulfidic Waters

As a fingerprint of the resistance of colloids against redox-driven dissolution, Brendel and Luther^287^ detected Fe(III) colloids in anoxic sediment porewaters. In a recent study, Engel et al.^192^ found that despite anoxic conditions (dissolved oxygen <0.08 mg L^–1^) and the presence of dissolved Fe(II), a significant portion of the colloidal Fe in a redox-dynamic floodplain remained in its oxidized form as ferrihydrite. Indeed, complexation or coprecipitation of ferrihydrite colloids with OM coatings has been shown to prolong the persistence of ferrihydrite colloids in an anoxic aqueous phase.^247,288−292^ Engel et al.^192^ reported that factors favoring the delay/inhibition of redox transformation likely include (1) high OM/Si loading and resulting coverage of the Fe-(oxyhydr)oxide surface,^220,293−295^ (2) the presence of OM-Fe(II) complexes in the aqueous phase, which can bond and stabilize Fe-(oxyhydr)oxide,^296^ and (3) surface organic functional groups—particularly the extent and type of associated ligands.^288,297−299^ Inversely, complexation of organic C on the surface of high surface charge colloids can potentially limit bioavailability of organic C, while being transported through a watershed.^29^

#### 4.3.4 Colloid-Facilitated Transport of Insoluble Redox-Sensitive Nutrients/Contaminants

Nutrients and contaminants that are relatively insoluble in their reduced forms (e.g., U, Cr, V, Se, Pu, Tc)^300^ are regarded as immobile under anoxic conditions. However, insoluble products can be mobile in the aquatic environment if they are present as colloids. For example, U(IV)-silica colloids have been observed at circumneutral pH,^301^ while U(IV) is relatively insoluble. More specifically, colloids precipitating from relatively insoluble reduced nutrients/contaminants could be small enough to transport in reduced aqueous environments. Indeed, Suzuki et al.^302^ showed that uraninite (UO_2_) colloids formed from bacterial reduction of U in sediments are typically of less than 2 nm diameter. Thus, precipitation of U as insoluble uraninite does not necessarily lead to complete immobilization. Hence, to predict the likely environmental fate of UO_2_ nanosized colloids, it will be necessary to determine the transport physics of isolated nanosized colloids and of those sorbed to larger colloidal particles and organics, and to define models that link crystallization with crystal-growth kinetics, aggregation kinetics, and fluid flow.^302^ Further, NOM and Fe-NOM has been shown to complex with sparingly soluble Cr(III) under anoxic conditions (see section 4.2.2) (e.g., refs (38 and 303−305)).

## 5 Experimental Challenges and Needs for Measuring and Characterizing Colloids and Colloidal Transport in Redox-Dynamic Environments

Although redox influences on the generation (see Section 3), behavior (chemical transformation and aggregation; see Section 4), and subsequent transport of natural colloids have been clearly demonstrated, the colloidal aspects of redox dynamics are difficult to characterize in natural environments and field samples. Most of the existing research on colloid transport affected by redox environments has been observational, with the key model parameters (e.g., kinetic rate of generation, dynamic surface area) still poorly constrained and unquantified. This lack of knowledge is compounded by challenges associated with the detection and characterization of colloids in natural redox environments, as well as the lack of systematic experimental setups to obtain necessary data to parametrize and validate models to account for colloids.^306^

### 5.1 Operational Challenges Due to Lack of Systematic Size Definition of a Colloid

As discussed in Section 2.1, the lack of a unified size definition for colloids makes comparison across studies difficult. Most common sampling approaches ignore the presence of colloids, their chemical transformation/aggregation, and their transport. Many studies consider certain colloidal nutrients and contaminants in the aqueous phase to be “*dissolved*”, based on the utilized filtration membrane pore size.^169^ Even when colloids are considered, their size range changes from study to study and specific ranges of colloidal sizes may still be excluded depending on the study and methodological approach taken and the research question targeted. As noted by Bao et al.,^169^ many studies use a 450 nm filter to separate “solid particles” from “colloids”,^163,173,307^ whereas other studies use the same filter size to separate “colloidal” (<450 nm) from “dissolved” (<450 nm) species.^308^ Further, considering that “*in situ*” sampling equipment typically used to collect porewaters have orifices smaller than 1000 nm (150 and 600 nm rhizons being the most commonly used for *in situ* sampling in soils and sediments)^192^ and larger colloids are often disregarded. Finally, while Bao et al.^169^ and numerous other studies define truly dissolved fractions as all material below 3 kDa, this excludes sulfide aqueous clusters observed by Luther and Rickard^66^ (and discussed in detail earlier in Section 2.1). These operationally defined size limits for “colloids”^163,173,307^ are arbitrary from a functional perspective and can significantly impact our understanding of the transport and bioavailability of nutrients and contaminants that may be associated with colloids.

### 5.2 Locating Redox Changes and Their Temporal Variability in Natural Conditions

The majority of studies on colloids have been performed using either laboratory-synthesized colloids^173,249,254^ or lab-simulated experiments using colloids extracted from soils/sediments.^29,200^ These lab experiments allow researchers to constrain the system and focus on a limited number of parameters. However, they are not necessarily representative of pore, ground, and surface water colloids, which are expected to exhibit pronounced compositional complexity.^192^ Thus, understanding and simulating colloid transport affected by redox-dynamic environments requires, retrospectively, data comparison of lab-simulated experiments with natural conditions in order to validate the lab-simulation results and constrain future lab and numerical simulation experiments.

However, like most soil properties and processes, redox is spatiotemporally heterogeneous.^309^ Important questions still remain regarding where exactly redox-generated colloids are promoted in soils and sediments, and how best to track the potential stability of these colloids to chemical transformation and aggregation during colloidal transport. Because redox spatiotemporal heterogeneity has not yet been incorporated into mainstream conceptualizations of soil biogeochemistry,^309^ these questions remain unresolved for most environmental systems, affecting our ability to *in situ* track redox-generated colloids, monitor their behavior and transport, and fully understand the impacts on nutrients and contaminants associated with colloids in redox-dynamic environments. To address this knowledge gap, Lacroix et al. recently published a review where authors describe past and current approaches for detecting, quantifying, and characterizing redox heterogeneities in soils as a function of time.^310^ The recent development of 2D imaging techniques, such as planar optode measurements, spatially resolved microbial techniques, and X-ray fluorescence imaging combined with spectroscopy, are promising and provide reliable forensic evidence for future research that integrates the influence of redox heterogeneities in generation, stability, and transport of colloids in soils and sediments.

The temporal dynamics of redox conditions are mainly driven by hydrologic regimes (e.g., snowmelt, precipitation, drought) that in turn induce changes in colloidal physicochemical composition. Thus, for the same location, the generation mechanisms of colloids, their physicochemical transformations and stability against aggregation, should also be tracked as a function of time/season and the changing of surrounding physicochemical parameters. However, the kinetics of redox changes are still poorly understood in natural environments. Thus, the time window needed between sampling events to observe changes in the nature and physicochemical composition of colloids remains unspecified and will likely differ for different sites.

### 5.3 Preservation of Redox Integrity

In addition to the lack of systematic and consistent colloid consideration/definition, the physicochemical properties of colloids in a natural aqueous phase can easily change during sampling, storage, and analyses, due to aging, changes in pH, ionic strength, redox conditions, and light exposure.^311,312^ Furthermore, associated nutrients and contaminants are also affected by sorption processes, complexation, and redox precipitations that can occur during transport and storage.

Characterizing suboxic/anoxic environmental colloids requires careful protocols for both sampling and analysis to preserve the native redox status of targeted elements. Unintentional introduction of O_2_ can lead to the oxidation of pore, ground, or surface water samples; for example, oxidation of dissolved Fe(II) can form nanosized Fe-(oxyhydr)oxide colloids, which may not be naturally occurring in the sampled aquifer.^125^ Such oxidation artifacts not only affect the physicochemical properties of colloids but could also impact the speciation of associated elements, such as phosphorus that may readily adsorb to nanosized Fe-(oxyhydr)oxide colloids^313^ (as also discussed in Section 3.2.1). Consequently, sampling potentially anoxic solutions requires systematic collection and analysis methods in order to maintain redox and chemical integrity throughout the sampling-analysis continuum. For example, a study by Dai et al.^314^ performed groundwater sampling and cross-flow ultrafiltration analysis with deliberate redox control measures and found no significant association of Pu with colloids in groundwater at the U.S. Department of Energy Savannah River Site, which is in contrast to a similar study at the same field site with no redox control measures, where investigators found a significant amount of Pu association with colloids.^315^ However, sampling anoxic waters requires proper training on elaborate techniques that are costly, difficult to obtain, and rarely tested for accuracy.

Preserving the redox state in collected samples is also a complicated task, particularly in the field. For example, even if oxygen is eliminated, the reduction reaction, if still active, could produce new colloids or change physicochemical properties of existing colloids after sampling, even if collected carefully. Most preservation approaches consist of freezing samples, whether by slowly cooling down to freezing temperatures^316^ or flash-freezing (L-N_2_) the samples immediately.^317,318^ However, the freezing process itself can facilitate irreversible precipitation of salts,^319^ the formation of coatings on colloids,^320^ and/or colloid flocculation,^311^ altering the colloid concentration and requiring further consideration during analysis. Although, temperatures just above the freezing point (e.g., commonly used 2–5 °C) are believed to minimize reaction kinetics, these have not yet been systematically tested for their efficiency in halting the biogeochemical reactivities completely, particularly in anoxic environments. The lack of systematic and consistent redox preservation approaches could impact the accuracy of the biogeochemical parameter libraries used for simulating colloid transport.

Nonetheless, some general practices have been pointed out as being helpful to consistently apply while preserving natural colloid samples, for instance, minimizing exposure to light, maintaining low temperatures, and minimizing physical disturbance once collected, choosing materials (tubing, vials, etc.) that are known for restricting O_2_ penetration and have lower affinity sorbent walls. If dilutions are absolutely necessary, maintaining pH, ionic strength, and ionic composition is critical.

## 6 Current State of Colloidal Transport Modeling and Associated Gaps Accounting for Redox

Based on the preceding sections, it becomes clear that effective colloid modeling requires the integration of several key processes: the generation of colloids in redox-dynamic environments, the stability of colloids as influenced by various chemical factors, and the transport mechanisms of colloids within different environmental systems. Additionally, colloid transport processes are inherently dynamic, varying both temporally and spatially. Specifically, generation/detachment (i.e., source) and/or retention/size exclusion (i.e., sink) processes are heavily influenced by spatial and temporal variability of unsaturated/saturated and redox conditions (see Sections 3 and 4),^321−327^ which are highly heterogeneous in soils and sediments.^51,309^ Contrasting conditions found in saturated and unsaturated porous media necessitate distinct approaches, which present additional challenges for modeling colloid transport.^5^ Similarly, redox variability can significantly change chemical conditions along a flow-path and/or over time (e.g., during wetting/drying or in relation to microbial metabolic activity).

In this context, addressing these challenges requires a thorough understanding of how redox fluctuations occur in the subsurface and the factors contributing to these variations. This comprehensive understanding is pivotal in guiding the design of experiments for data collection, an essential step for developing robust reactive transport models (RTMs) that accurately and effectively represent environmental systems. The following sections present a concise overview of colloid transport continuum-based numerical models, aiming to highlight the capabilities and limitations in RTMs in effectively incorporating redox dynamics.

### 6.1 Modeling Colloid Transport in Porous Media under Dynamic Redox Conditions

Colloid Filtration Theory (CFT) in porous media, the process of removing suspended particles from a fluid by passing it through a porous medium such as a filter or a bed of particles,^206,328^ has played a significant role in the development of these colloid transport models and approaches for understanding and predicting colloid behavior in porous media. To describe the partitioning of colloids between the aqueous-phase and solid-phase matrix in variably saturated porous media, a general advective dispersion equation (ADE) for colloid transport is used (eq 1):
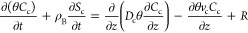
1

The left-hand side terms account for the temporal variations of both immobile (*S*_c_ [M M^–1^]) and mobile (*C*_c_ [M L^–3^]) colloids on the solid surface of a porous solid-phase matrix; θ [L^3^ L^–3^] is the volumetric water content, ρ_B_ [M L^–3^] is the bulk density, and *t* [T] is time. The right-hand side terms represent the flow of colloids through the pores of the porous solid-phase matrix, where *v*_c_ [L T^–1^] and *D*_c_ [L^2^ T^–1^] are the advective (i.e., Darcy or pore-water) velocity and the dispersion coefficient for colloidal particles, respectively; *R* describes any additional source or sink terms of colloids which are not integrated in the rest of the equation.

The presence of uncertain terms, such as *R*, within eq 1 presents a valuable opportunity for advancing the integration of redox dynamics into colloid transport by explicitly incorporating them into the equation. For instance, source and sink terms account for colloid generation and retention. However, distinguishing whether these processes are primarily driven by attachment or size exclusion mechanisms within the existing modeling framework is often challenging. The following section will explore these concepts in more detail and identify potential avenues for integrating redox dynamics with colloid transport.

#### 6.1.1 Modeling Colloid Attachment in Porous Media

Commonly, quasi-first-order rate coefficients, or composites of rate coefficients, are used to model the partitioning of colloids between the surfaces of porous solid-phase matrix (stationary) and aqueous phases, via the attachment mechanism described by CFT.^329,330^ The rate of change in the concentration of immobilized colloids due to attachment can be modeled using first-order kinetics as follows (eq 2):
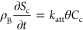
2

The attachment rate constant (*k*_att_ [T^–1^]), representing the rate at which colloidal particles deposit onto a porous media or soil/sediment solid-phase matrix, is directly influenced by two factors (eq 3): (i) the frequency of contact between colloids and the grain surface and (ii) the affinity of the colloids to adhere to the porous medium.^206,328^ The attachment rate constant is typically calculated as a function of the median radius of the grain of porous media (*a*_c_ [L]), Darcy velocity (*v*_c_ [L T^–1^]), porosity of the porous media (ε [−]), and collision (η [−]) and sticking (α [−]) efficiencies. These factors account for the diffusion, attachment, and gravitational sedimentation of colloidal particles.
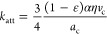
3

The attachment of colloidal particles is contingent on the presence of available attachment sites, which are influenced by factors like characteristics (e.g., surface area, charge, roughness) of the solid surface of the solid-phase matrix and colloid properties (e.g., functional groups). The Derjaguin–Landau–Verwey–Overbeek (DLVO) theory^331,332^ proposes that an interplay occurs between attractive van der Waals and electrostatic forces (either attractive or repulsive depending upon the charges between the colloids and surfaces), and this interaction is dependent on the distance between colloids and the surface. The DLVO theory not only considers the impact of van der Waals forces and electrostatic repulsion/attraction but also takes into account additional factors, such as Brownian motion (the random motion of particles suspended in a fluid) and the ionic strength of the surrounding aqueous phase. Redox reactions significantly impact surface charges (see Section 4.1.3) and thus the prevalence of both favorable and unfavorable conditions conducive to colloidal attachment (i.e., the attachment efficiency, α). Furthermore, redox processes can also shift the parameters (pH, ionic strength, and ionic composition; see Section 4.1) and complexing agents (see Section 4.2) of the aqueous phase that govern colloid attachment to the solid surfaces of a porous solid-phase matrix. For instance, the generation or release of colloids with a high adsorption capacity (such as Fe-(oxyhydr)oxide, clays) can trap dissolved and particulate NOM in suspension (see Section 4.2) and reduce the attachment efficiency of NOM to a porous solid-phase matrix. Fully encapsulating all of the effect of these chemical processes into a single attachment efficiency term, α, is a challenge for current RTMs; thus, we recommend utilizing α terms that properly reflect changing particle and solution conditions.

#### 6.1.2 Modeling Colloid Size Exclusion in Porous Media

While CFT can reasonably estimate the collision [η] under conditions favorable for attachment, CFT does not work well under unfavorable conditions.^327,333,334^ Nonetheless, under unfavorable conditions, size exclusion has been suggested to account for filtration through porous media, including straining, wedging, and entrapment of colloids.^335−339^ Although these mechanisms remove colloids from the aqueous phase, they differ fundamentally from attachment mechanisms as they are primarily governed by geometrical constraints rather than electrostatic forces. However, there is a clear connection between the biogeochemical mechanisms, including redox and shifts in ionic strength, pH, and chemical composition, that physically change the shape and size of colloids (see Section 4.1), the collision efficiency, and size-exclusion effects.

While attachment removes colloids through collision, straining involves the filtration of colloids based on their size or shape. Similarly, wedging or entrapment involves trapping colloids between two surfaces enclosed within a larger solid aggregate or solid-phase matrix in porous media. A few studies have shown colloidal removal using both attachment and straining mechanisms.^337,339,340^ In particular, Bradford and Bettahar^335^ and Xu et al.^337^ used a dual-site approach to explain colloidal retention mechanisms. One site was dedicated to attachment, while the other focused on straining. They suggested that attachment is reversible, but straining is irreversible. They further suggested modifications to eq 1 to include two sites for colloid retention in numerical representations:
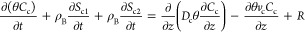
4

In the given context, the variables *S*_c1_ and *S*_c2_ represent the respective masses of colloids that have become attached to the solid-phase matrix and the masses of colloids that have been strained out of the aqueous phase. Furthermore, these authors aimed to model straining as a first-order kinetics process by determining the straining rate coefficient based on the distance calculated from the entrance of the porous solid-phase matrix.

Although the dual-site model approach is reasonable for simulating field scale colloidal transport processes, additional partitioning between attachment/detachment and straining fractions remain to be experimentally determined. Consequently, the dual-site approach has primarily been utilized in modeling studies where breakthrough curves can be calibrated. Future research is needed to calibrate colloidal behavior in broader applications, particularly accounting for dynamic redox conditions and associated physicochemical properties of colloids, aqueous-phase, and solid-phase matrices.

As discussed above, although different mechanisms (e.g., attachment, straining, entrapment) remove colloids from the system, they may yield different outcomes under prevalent redox conditions. Hence, understanding the specific role of redox on attachment, straining, wedging, or entrapment becomes crucial. Furthermore, redox conditions can alter the soil matrix through mineral dissolution (see Section 3.1), significantly affecting wedging or entrapment. This presents a valuable opportunity to expand and incorporate these mechanisms within RTMs while considering the influence of redox conditions.

#### 6.1.3 Modeling Colloidal Detachment in Porous Media

Detachment (as opposed to attachment) results in the separation of colloids from one another and/or from the porous solid-phase matrix. The detachment of colloids from the porous solid-phase matrix is the consequence of several physical factors, including hydraulic transients (such as infiltration and wet/dry cycles),^327,341^ colloid–colloid collisions,^48^ an increase in shear stress,^342−344^ and movement of the air–water interface.^345^ On the other hand, chemical perturbations such as redox changes and subsequent shifts in aqueous-phase chemistry (e.g., ionic strength, pH, chemical composition; see Section 3), surface properties, and electrostatic interactions, may further promote detachment due to the weakening of bonds between colloids and the porous solid-phase matrix.^5,339,346−351^
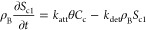
5

Moisture content^327,341^ is a key parameter in modeling colloidal detachment, as demonstrated in eq 5, where S_c1_ [M M^–1^] and *C*_c_ [M L^–3^] are the concentrations of colloids in the porous solid-phase matrix and mobile phases, respectively.^327,341^ Colloid detachment (*k*_det_ [T^–1^]) is determined by porewater velocity above a critical moisture content. Various studies^1,170,352−358^ have found that DLVO forces, which balance van der Waals attractive forces with electrostatic repulsive forces, control colloidal detachment. Thus, we highly recommend improving DLVO models by incorporating the biogeochemical perturbations affecting surface properties and electrostatic interactions, such as changes in redox conditions.

#### 6.1.4 Modeling Colloidal Generation in Porous Media

Research indicates that erosion processes have the potential to generate colloids. These newly generated colloids may not have originally existed in the dissolved phase but were removed from the porous solid-phase matrix.^343,359^ Researchers have categorized attached colloids into two distinct groups: those that are irreversibly attached and those that are attached but capable of being released. This categorization conceptually parallels eq 5, where attachment and straining are modeled separately. Additional terms representing colloid generation through physical detachment can be added to the equation as
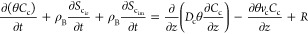
6where *S*_c_ir__ and *S*_c_im__ are irreversibly attached colloids and immobile colloids available for release, respectively.

In addition to erosion, colloids can be generated due to various other factors, including fluctuations in redox conditions and resulting changes in aqueous-phase chemistry, such as pH and ionic strength (see Section 4.1). However, colloid generation through redox variation is currently not well parametrized in models, primarily because more quantitative empirical evidence is needed. Most of the existing research on *in situ* release and new precipitation has been qualitative observational studies, and quantitative models that incorporate kinetics, dynamic surface area, and thermodynamic constraints are still lacking. The balance between *in situ* release and new precipitation is challenging to determine, especially for changing redox conditions, necessitating new experimental designs.

#### 6.1.5 Retention of Colloids via Attachment and Straining at the Air–Water Interface

In variably saturated porous media, colloids experience a wide range of spatially and temporally varying physical and chemical conditions.^105,360^ These factors significantly influence colloid transport, making it more intricate in unsaturated media compared to saturated media.^321^ Moreover, several processes occur in variably saturated porous media that do not occur in saturated conditions, such as discontinuous capillary fringes that promote colloid deposition or wetting–drying cycles that promote colloid transport.^322,323^ Overall, unsaturated conditions lead to a higher rate of colloid removal than saturated conditions.^324−327^ In addition, variations in subsurface redox states can alter solid-phase properties,^327,341^ surface charge, and microbial activity, ultimately impacting colloid behavior during these hydrological processes.

Moreover, redox states have the capacity to modulate the air–water interface by altering the local surface potential, consequently affecting the behavior of colloids at this interface.^361^ For example, under unsaturated conditions, colloids can attach to the air–water interface and undergo straining. This attachment is governed by electrostatic, hydrophobic, and steric interactions, and the resulting colloidal monolayers or multilayers can significantly impede colloid transport. Wan and Wilson^362^ found that colloids preferentially deposit at the air–water interface. This interface acts as a potent sorbing phase that significantly retards colloid movement in porous media. Hence, it is crucial to incorporate this phase into the modeling framework, for example, by introducing a Γ [L^–1^] term into eq 1, to represent the interfacial surface area (eq 7)
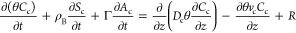
7where *A*_c_ [M L^–2^] is colloid concentration at the air–water interface.

Thin water films, referred to as film straining, pose another physical limitation on colloid transport in porous media under unsaturated conditions. This phenomenon has been extensively studied by Wan and Tokunaga,^345^ who suggested that colloid transport in unsaturated porous media is a function of the ratio between the size of colloids and the thickness of the water film. In addition, the air/water/solid-phase matrix contact line, which is formed by water molecules adsorbed onto the surfaces of colloids, has been observed to exert a noteworthy influence on the retention and release of colloids.^327,344,363,364^ Based on the previously established eq 8, Corapcioglu and Choi^338^ developed a numerical model to include colloid transport at the air/water/solid-phase matrix contact line into the modeling framework (eq 8). Here, *F*_c_ [ML^–3^ ] represents the concentration of colloids trapped by film straining.
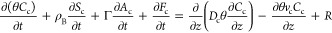
8

Importantly, redox states can influence colloid behavior at the air–water interface through various mechanisms. It is crucial to understand the processes at this interface, including its modulation, which can be shaped by local surface properties influenced by redox conditions (see Section 4). These alterations can have implications for attachment, straining, and related phenomena. For example, attachment at the air–water interface may be governed by electrostatic conditions and redox states. Likewise, as discussed earlier in Section 4.2.1, straining is a unique process that can impact colloid transport. These processes should be quantified under varying redox conditions to enhance our comprehension, offering the possibility of a transformative shift in colloid transport modeling.

### 6.2 Parametrization of Colloid Generation and Behavior

Colloid generation, *in situ* release, and newly precipitated colloids (see Section 3) have not been parametrized enough in numerical models and lack empirical data (see Section 5). Furthermore, the distinction between *in situ* release and new precipitation is difficult, especially under changing redox conditions. The development of lab-simulation experiments, using a stepwise process from simple batch experiments to more complex column experiments that are capable of mimicking distinct colloid-generating redox processes and allow transport considerations, is crucial for capturing the key parameters needed to model redox-generated colloids in porous media.

Once colloids are generated, their transport is dictated by their ability to interact with other colloids and the surrounding porous media, which is dependent on surface properties and electrostatic interactions. These surface- to pore-scale processes are poorly constrained for colloids. Thus, transport should be evaluated in column experiments with both well-constrained pore structure and material in order to identify the controlling mechanisms and kinetics.^365−367^ The transport of each well-characterized, monodisperse redox-generated colloid should be tested against a conservative tracer to calibrate transport properties. However, this approach complicates identifying distinct key parameters that are needed for modeling size exclusion (see Section 6.1.2) and attachment/detachment in porous media (see Sections 6.1.1 and 6.1.3). Specifically, thanks to the development of new imaging technologies, such as microfluidics and μ-X-ray fluorescence mapping, we suggest that future studies focus on understanding the influence of biogeochemical processes on the partitioning between size exclusion as a consequence of aggregation mechanism and attachment conditions.

### 6.3 Simulating the Impact of Colloidal Transport in Redox-Dynamic Environments

The challenges previously described in detecting and characterizing colloids associated with redox-dynamic environments in natural conditions go hand in hand with major challenges in integrating necessary data to parametrize and validate models to account for colloids at field scale. Thus, additional knowledge is needed about the relevance of colloidal transport and exported reactivity for ground and surface water quality and for RTMs to include colloidal transport at the field scale. As an example, Babey et al.^172^ showed, through RTM simulations based on data from a series of dual-domain column experiments,^259,260,368,369^ the necessity to consider transport of NOM colloids from anoxic environments as the key driver to explain the observed biogeochemistry downstream in an initially oxic surrounding environment. Indeed, the NOM colloidal transport from anoxic lenses to the oxic surrounding sediment drives the development of proximal secondary reduction zones (“halos”), characterized by high microbial activity (e.g., sulfate reduction) and accumulation of reduced reaction products (e.g., iron sulfide).^22,370^ This study corroborates that neglecting colloidal transport within and from redox-dynamic environments creates major uncertainties in model simulations of pore, ground, and surface water quality. Future research should focus first on developing RTMs to test if colloidal transport could explain some paradoxical/unexpected field-scale nutrient and contaminant cycling in redox-dynamic environments.

## 7 Conclusions and Future Recommendations

This review emphasizes the presence and role of colloids in the transport of nutrients, contaminants, and organic matter through environmental systems. As consistently highlighted in this review, colloid generation and transport mechanisms are not yet clearly understood and even less quantified/parametrized, partly due to methodological challenges in sampling and analyses of colloids in natural systems, especially redox-dynamic ones. However, it is only logical to anticipate that generation, behavior, and transport of colloids under redox dynamic environments can be different than those under stable oxic environments. Therefore, colloids in redox-dynamic environments have yet to be part of routine sampling protocols or general predictive geochemical modeling approaches.

A major challenge in colloid research is the lack of a universal definition of a “colloid”, which leads to inconsistencies in identification and characterization of colloids in field- and lab-scale studies. Here, we propose a pragmatic approach toward nomenclature of colloids that could bring more uniformity, clarity, and consistency in colloidal research. We propose broadening the size range to cover various environmental colloids and inclusion of functionality in the definition of colloids. Being aware of biogeochemical conditions of the system that are conducive to colloidal generation will allow researchers to adopt a tailored sampling plan (frequency as well as key parameters) during an experiment. Additionally, attention is needed to address the lack of consistent experimental approaches for analyzing colloids in samples from potentially anoxic environments. We propose greater clarity in communicating how various methodological approaches, from sampling to analyses, may impact the results of colloid studies in redox dynamic environments, including the implications on what is considered a colloid.

While similar solution parameters (pH, ionic strength and composition, solid/solution ratio) control colloidal generation and stability in oxidized or reduced environments, the complexity is accentuated in redox-dynamic environments, where redox reactions can *in situ* catalyze sudden changes in solution chemistry that influence colloidal behavior. Consequently, colloidal transport-behavior relationships are challenging to predict, necessitating further model development to gain a deeper mechanistic understanding. To be able to study these reactions, sampling plans need to be spatially and temporally elaborate. This has implications for the mass balance of elements (trace metals, nutrients) and colloid-facilitated transport of contaminants. In addition, in anoxic environments, where results are more easily compounded by artifacts due to the materials used in sample collection (tubing, filter material, etc.), preservation methods and the ability of analytical techniques to maintain redox integrity during analysis are pertinent. In sum, there is need for tighter coupling between field, lab, and modeling studies to increase the efficiency and accuracy of data interpretation and knowledge on the environmental role of colloids, particularly in redox-dynamic systems.

In this review, we have examined existing literature on natural colloids and their environmental importance, with a focus toward redox-dynamic systems. We have identified and discussed challenges and opportunities related to the consideration of colloids in routine analyses, focused studies, and biogeochemical models. Our intent and hope is that this work will lead to broader and more open discussions that will address these issues, including workshops and hands-on training on sampling, analytical, and experimental approaches, as well as modeling tools that will help usher the research and understanding of colloids in environmental systems.
